# Point-of-care platelet function tests: relevance to arterial thrombosis and opportunities for improvement

**DOI:** 10.1007/s11239-020-02170-z

**Published:** 2020-06-11

**Authors:** Diana A. Gorog, Richard C. Becker

**Affiliations:** 1 National Heart and Lung Institute, Imperial College, Dovehouse Street, London, SW3 6LY UK; 2 University of Hertfordshire, Hertfordshire, UK; 3 University of Cincinnati College of Medicine, Cincinnati, USA

**Keywords:** Platelet aggregation, Platelet function test, Thrombosis, Fibrinolysis, Citrate, Shear

## Abstract

Studies using whole blood platelet aggregometry as a laboratory research tool, provided important insights into the mechanism and modulators of platelet aggregation. Subsequently, a number of point-of-care (POC) platelet function tests (PFTs) were developed for clinical use, based on the concept that an individual’s thrombotic profile could be assessed in vitro by assessing the response to stimulation of platelet aggregation by specific, usually solo agonists such as adenosine diphosphate (ADP), collagen and thrombin. However, adjusting antiplatelet medication in order to improve the results of such POC PFTs has not translated into a meaningful reduction in cardiovascular events, which may be attributable to important differences between the POC PFT techniques and in vivo conditions, including patient-to-patient variability. Important limitations of most tests include the use of citrate-anticoagulated blood. Citrate directly and irreversibly diminishes platelet function and even after recalcification, it may result in altered platelet aggregation in response to ADP, epinephrine or collagen, and interfere with thrombin generation from activated platelets. Furthermore, most tests do not employ flowing blood and therefore do not assess the effect of high shear forces on platelets that initiate, propagate and stabilize arterial thrombi. Finally, the effect of endogenous thrombolysis, due to fibrinolysis and dislodgement, which ultimately determines the outcome of a thrombotic stimulus, is mostly not assessed. In order to accurately reflect an individual’s predisposition to arterial thrombosis, future tests of thrombotic status which overcome these limitations should be used, to improve cardiovascular risk prediction and to guide pharmacotherapy.

## Highlights

- Commonly used point-of-care platelet function tests have many limitations and do not accurately reflect the in vivo situation.
- To assess overall thrombotic status, a global thrombotic stimulus (like high shear) should be used, rather than assessing the response to individual agonist(s) (ADP, collagen, thrombin).
- Native blood is preferable to citrated blood, as the latter may alter platelet aggregation and thrombin generation.
- Accurate thrombotic status assessment should also assess endogenous thrombolysis.
- Tests of thrombotic status which overcome these limitations may better reflect physiology, improve cardiovascular risk prediction and help tailor pharmacotherapy.

## Introduction

The discovery that platelet aggregation is inducible in vitro*,* and the development of an instrument to measure aggregation quantitatively, began a flourishing era in thrombosis research [1–3]. Platelets are highly reactive cells and aggregation can be induced by a variety of substances including chemicals, proteins, foreign surfaces and altered flow conditions. Among these, collagen, adenosine diphosphate (ADP) and thrombin-induced platelet aggregation generated greatest interest due to their physiological significance and potential use for the development of targeted therapies. As platelet aggregometry evolved, from testing platelet-rich plasma to whole blood, and replacement of a chart recorder with automated recording, it gained popularity in clinical research. While aggregometry has remained an extremely useful laboratory tool, point-of-care (POC) instruments such as the platelet function analyser (PFA-100/PFA-200; Siemens Diagnostic Deerfield, Illinois, USA), VerifyNow (Werfen, Barcelona, Spain), Plateletworks (Helena Laboratories Beaumont, Texas, USA), TEG the thrombelastography (TEG; Hemoscope Corporation, Niles, Illinois, USA), the rotational thromboelastometry (ROTEM; Werfen, Barcelona, Spain) [4–6], and more recently the Global Thrombosis Test (Thromboquest Ltd., London, UK) [7] have been increasingly used in clinical settings. Early POC techniques were considered highly pathologically-relevant, since the release of agonists, such as ADP, from activated platelets was regarded as the single most important mechanism initiating arterial thrombosis. However, having been used in a number of clinical studies for more than a decade, many early POC techniques have not found a use in routine clinical care and may be considered as research tools with limited clinical utility*.*[8, 9]. Despite strong evidence for an association between persistent high on-treatment platelet reactivity (HTPR) and an increased risk of adverse cardiovascular events, several large studies using POC techniques did not show more favourable clinical outcomes when antithrombotic treatment was either altered or titrated to reduce platelet aggregation using these techniques, and therefore POC platelet function tests (PFTs) are currently not recommended for routine clinical use [10–13]. In most such trials, POC PFTs did not improve clinical outcome compared to standard antiplatelet therapy without monitoring [14, 15]. More recently, POC PFT have been used to support de-escalation of antiplatelet therapy in patients with acute coronary syndrome (ACS). In elderly ACS patients, similar ischaemic and bleeding rates were observed whether patients were treated with conventional low dose prasugrel (5 mg/day), or with PFT-guided prasugrel escalation to 10 mg daily or de-escalation to clopidogrel [16]. Amongst patients with ACS treated with PCI, PFT-guided de-escalation from prasugrel to clopidogrel was non-inferior to standard prasugrel with respect to net clinical benefit (combined ischaemic and bleeding events) at 1 year [17]. However, these PFT-guided de-escalation studies were underpowered to assess safety with respect to major clinical events such as myocardial infarction and stent thrombosis, so this approach cannot be adopted into clinical care as assumed safe practice.

It is important to consider why POC-guided therapy failed to translate into a meaningful reduction in major adverse thrombotic events. [9, 18]*.* One can postulate that this was due to either trial design, assessment of low risk populations, variability in the HTPR definition and possible failure to adequately overcome HTPR. However, it is also possible that POC PFT lacked direct pathological relevance to arterial thrombosis in vivo (Table 1). This review will focus on pathologically-relevant issues pertaining to the assessment of arterial thrombosis and consider how future tests could be tailored to more accurately reflect clinical conditions, in order to improve the assessment of thrombotic risk and personalize pharmacotherapy.

**Table 1:** Comparative features of point-of-care tests of platelet function

Instrument	Manufacturer	Technique	Anticoagulant	Agonist used to stimulate thrombus formation	Assesses contribution of RBC/WBC	Flowing blood	Shear*/gradient	Assesses endogenous thrombolysis/fibrinolysis
PFA-100 PFA-200	Siemens Healthcare GmbH, Erlangen, Germany	Measures closure time (occlusion of a coated membrane aperture)	Citrate	Three assays with different agonist: Collagen/EPI assay, Collagen/ADP assay, and Innovance P2Y test	No	Yes Low flow	Sub-pathologic ~ 5000 s^−1^	No
VerifyNow	Werfen, Barcelona, Spain	Measures optical signal of light transmitted through whole blood which depends on the degree of platelet aggregation around fibrinogen coated beads	Citrate	Three assays vary in platelet agonist: -Aspirin assay uses AA -P2Y12 assay uses ADP -Gp IIb/IIIa assay	No	No	No	No
Plateletworks	Helena Laboratories Beaumont, Texas, USA	Measures platelet count before and after addition of platelet agonist	None in agonist tubes, but EDTA in reference tube	ADP, Collagen or AA agonists	No	No	No	No
Impact R	DANED SA, Beersel, Belgium	Cone and plate viscometry- Measures platelet inhibition and aggregation induced by extracellular matrix and very low shear stress	Citrate	Extracellular matrix	Yes	Yes	Sub-Pathologic ~ 1800 s^−1^	No
Multiplate	Roche Diagnostics, Basel, Switzerland	Impedance aggregometry	Hirudin	ADP test, ASPI test (AA), TRAP test	No	No	No	No
TEG/ROTEM	TEG; Haemonetics Corporation, Braintree, MA, USA ROTEM; Werfen, Barcelona, Spain	Measurement of whole blood clotting (not platelet function), in response to rotation and measurement of viscoelastic clot characteristics reflected by changes in impedance	Citrate	Low shear and external agonists (eg kaolin, ellagic acid, tissue factor) More akin to venous thrombosis/clotting than arterial thrombosis	Yes	No	No shear 0.1 s^−1^	Clot lysis, not thrombolysis or fibrinolysis
Global thrombosis test	Thromboquest Ltd, London, UK	Measurement of thrombus formation in response to high shear, and subsequent measurement of spontaneous thrombolysis	None	High shear only (no external agonists)	Yes	Yes	Pathologic ~ 12,000 s^−1^	Yes

*AA* arachidonic acid, *ADP* adenosine diphosphate, *EDTA* Ethylenediaminetetraacetic acid, *EPI* epinephrine, *Gp* glycoprotein, *RBC* red blood cells, *TRAP* thrombin receptor activating peptide, *WBC* white blood cells

*Pathological shear rate > 10,000 s^−1^

## Determinants of arterial thrombosis

**Fig. 1:**
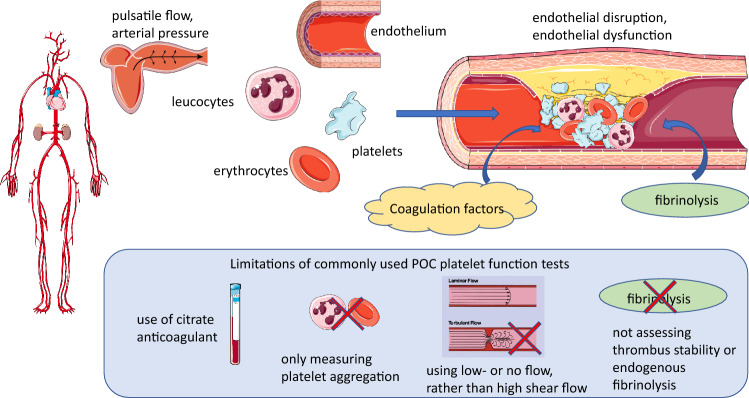
Main pathophysiological determinants of arterial thrombosis and limitations of current point-of-care (POC) platelet function tests. Arterial thrombosis under pathological conditions is driven by shear gradient-mediated platelet aggregation and activation of coagulation, resulting in an occlusive fibrin mesh, in which entrapped erythrocytes and leucocytes make important contributions to thrombus stability, as well as fibrinolysis. Rheological flow characteristics and effectiveness of endogenous fibrinolysis determine thrombus stability and lysis. Important limitations of many POC PFTs include (i) use of citrate-anticoagulated blood, which even after recalcification, may result in impaired platelet aggregation and thrombin generation from activated platelets, (ii) not assessing the contribution of erythrocytes, leucocytes and NETs, (iii) use of stagnant conditions or laminar blood flow and therefore not assessing the effect of high shear gradient on platelets and (iv) failing to assess the effect of endogenous thrombolysis

There are many components of arterial blood that determine thrombosis, some of which can be replicated in vitro (Fig. 1). Whilst thrombosis can clearly occur in an otherwise healthy vessel, the vast majority of local thrombus formation (in contrast to embolization) occurs in atherosclerotic arteries, with some degree of endothelial injury or dysfunction. Cellular components together with plasma proteases, specifically components of the coagulation and the fibrinolytic pathways, as well as the characteristics of blood flow impact on the development and stability of the growing thrombus. The ideal test to assess thrombosis should incorporate these features as much as possible, to simulate in vivo conditions.

### Cellular components

Platelets are the predominant mediator of arterial thrombosis, through platelet activation, adhesion and aggregation. Platelets contribute to both coagulation and thrombolysis, mainly through thrombin generation and release of plasminogen activator inhibitor 1 (PAI-1). Leucocytes and erythrocytes are important mediators of thrombus stability, which impart both structural stability as well as mediate resistance to fibrinolysis. Neutrophils release proteases such as elastase and cathepsin G, which exert a plasmin-independent fibrinolytic effect, including inactivating the major plasmin inhibitor α_2_-PI [19]. Such proteases can directly alter platelet function and/or participate in coagulation cascade reactions on the platelet or neutrophil surface to enhance fibrin formation. In addition, neutrophils contribute to thrombosis through the formation of neutrophil extracellular traps (NETs), which promote thrombosis by providing a scaffold to entrap platelets, erythrocytes, and procoagulant molecules, as well as enhancing coagulation through the intrinsic and extrinsic pathways [19].

### Coagulation and fibrinolytic pathways

Activation of coagulation is necessary for fibrin formation, which is essential for cross linking platelets within the thrombus, as well as the stimulation of fibrinolytic enzymes such as plasmin and antithrombin. Not only is it important to assess the pro-coagulant tendencies, but also to consider the effectiveness of endogenous thrombolytic/fibrinolytic pathways, which can determine the outcome of a pro-thrombotic stimulus [19].

### Haemodynamic conditions

The characteristics of blood flow, including laminar flow, pulsatility, arterial pressure and local haemodynamic/shear forces, impact the development and stability of the thrombus. Rheological disturbances have an important role in promoting occlusive thrombus formation, as thrombus propagation and vessel occlusion tend to occur on plaques causing marked disturbance of laminar blood flow, rather than on non-stenotic lesions [20]. In severely stenotic arteries, pathological wall shear rate can exceed 10,000 s^−1^, and may reach 100,000^–1^ [21]. High shear forces that occur in areas of accelerated flow at the site of maximal stenosis potentiate platelet activation, whilst the turbulence in the low-flow, low-shear, deceleration zone in the post-stenotic region provides an ideal flow environment for the progressive accrual and aggregation of platelets. At the same time, faster flow through the stenosis as well as flow pulsation also favour thrombus embolization.

### Endothelium

The healthy endothelium can exert antithrombotic effects, through release of prostacyclin and nitric oxide, potent inhibitors of platelet and monocyte activation. Endothelial cells can secrete tissue plasminogen activator, which breaks down fibrin, as well as von Willebrand factor (vWF), which can initiate platelet aggregation and contribute to blood coagulation [22]. While endothelial cells do not express much tissue factor (TF), they have important anticoagulant effects since they are the predominant source of TF pathway inhibitor (TFPI), as well as through surface expression of thrombomodulin, which binds thrombin, acting as a potent anticoagulant. Pathological arterial injury with plaque erosion or rupture results in expose of subendothelial contents including collagen, which activates the contact system, resulting in FXIa-driven thrombin generation, leading to platelet activation and TF that is a strong stimulator of the prothrombinase complex, thrombin generation and fibrin formation. Contact activation can also be induced by blood contact with negatively charged surfaces of biomaterials. Most patients at thrombosis risk exhibit endothelial dysfunction, which manifests in loss of protective molecules and expression of adhesive molecules, and increased secretion of PAI-1 and vWF.

## Limitations of commonly used point-of-care PFTs

Advances in research in the past few decades have shed light on the mechanism of occlusive arterial thrombosis, revealing important new determinants that were previously unrecognised, including altered flow conditions at high shear rates (shear gradients), local thrombin generation by shear-activated platelets, the contribution of coagulation factors and NETs, and the importance of fibrin clot stability and fibrinolysis. This new knowledge has relegated to secondary significance the role of specific agonist-induced platelet aggregation in mediating thrombus formation. However, these newer determinants of thrombosis are not reflected in or assessed by many of the POC tests commonly in use in clinical settings.

### Assessment of platelets function in whole blood

Whilst most tests use whole blood, some such as Platelet Works, the IMPACT: Cone and Plate(Let) Analyzer (Image Analysis Monitoring Platelet Adhesion Cone and Plate Technology) (CPA) (DANED SA, Beersel, Belgium) and Multiplate Analyzer (Roche, Basel, Switzerland) although employing whole blood, give results that reflect only platelet aggregation specific to a particular platelet agonist, and do not take account of the contribution of other blood constituents to thrombus formation.

### Use of a single agonist to initiate platelet activation

Many physiologically-relevant agonists (such as ADP, epinephrine, collagen) can cause platelet activation, adhesion and aggregation both in vivo and in vitro. However, in a severely stenosed artery, it is shear-gradient dependent platelet aggregation mechanism which drives thrombus formation [23–25] while soluble agonists released from platelets play a secondary role, mainly in stabilizing formed aggregates. Despite this, the most commonly used tests such as the PFA and the VerifyNow still employ single agonists such as ADP or collagen to initiate platelet aggregation to determine the likelihood of arterial thrombosis, such as the PFA-100 and the VerifyNow. These tests do not reflect arterial thrombogenesis under high shear conditions.

### Use of anticoagulant interferes with thrombus formation

#### Effect of citrate on ionized calcium concentration

Most POC PFTs in current clinical use are performed on anticoagulated blood. There are very few exceptions, notably the Global Thrombosis Test and Plateletworks, which employ native non-anticoagulated whole blood. Sodium citrate is the most commonly used anticoagulant for POC PFTs and for platelet aggregometry in platelet-rich plasma or in whole blood. Citrate acts by chelating extracellular ionized calcium (iCa^2+^), reducing the concentration in the blood sample from ~ 1.2 to ~ 0.1 mmol/L. Citrate anticoagulation is a very convenient way to handle blood samples, allowing storage for a few hours and transfer of samples from the patient to the laboratory for subsequent analysis. However, the “convenience” of anticoagulation comes at a price; namely the rendering of the blood sample highly non-physiological, such that it lacks relevance to the function of whole blood and platelets under thrombotic or haemorrhagic conditions in vivo. The serious drawbacks of citrate anticoagulation have been known for a long time, but there has been no viable alternative proposed and it has not been considered possible to overcome the logistic need for storage of blood prior to analysis. Citrate is added to blood in a fixed concentration and the final plasma iCa concentration after citrate is slightly above the threshold required for activation and aggregation of platelets without triggering blood coagulation. Citrate anticoagulation assumes that plasma iCa concentration in individuals is standard, but this is not the case [26]. Stratification of subjects based on iCa concentration identified that approximately 15% of subjects had a serum iCa above, 2% below, and only 69% had iCa within the normal range (1.12–1.30 mmol/L)*.*[27] Sustained hyper- or hypocalcaemic disorders have significant physiological effects that can contribute to onset and progression of cardiovascular disorders or bleeding, respectively.

Considerable evidence shows that hypercalcaemia is associated with an increased risk of developing cardiovascular disease, arterial thrombosis and death [28–30]. As extracellular iCa concentration determines the rate of platelet reactivity to various agonists [31], variation in plasma iCa concentration results in highly variable aggregation response to agonists, especially if the agonist is applied in submaximal concentration [32]. Other factors can also influence plasma iCa [26]. The inverse relationship between serum iCa concentration and parathyroid hormone levels is well established. In patients receiving dual antiplatelet therapy, higher parathyroid hormone levels were associated with an increase in ADP-mediated platelet reactivity. Dietary factors such as citrate consumption can cause transient hypocalcaemia. It is therefore possible that measured platelet reactivity may vary with factors affecting plasma iCa concentration, rather than simply reflecting pharmacological resistance to antiplatelet therapy.[10, 33]

#### Effects of citrate on coagulation and thrombus stability

Citrate anticoagulation directly impairs coagulation dynamics [34] and the activation and adhesion of platelets and leukocytes in a concentration-dependent manner [35]. Extensive aggregation of human platelets in response to ADP, followed by secondary aggregation due to the release of platelet granule contents, occurs only in citrated blood. The main agent responsible for ADP-induced secondary aggregation and granule release appears to be sodium citrate [36, 37]. In citrated blood, the extent of aggregation did not change for up to 2 h after blood collection, but subsequently dramatically decreased. It was therefore recommended that platelet aggregation studies should be completed within 2 h of sampling, instead of the current practice of 4 h [38]. Relatively small increments in citrate concentration markedly inhibited platelet aggregation in response to ADP, epinephrine or collagen, and the effects of these agonists were much more marked in citrate-anticoagulated than in hirudin/PPACK-anticoagulated blood [37, 38]. Furthermore, the use of citrate anticoagulant significantly impaired platelet aggregation compared to anticoagulation with a direct thrombin inhibitor or unfractionated heparin, even when platelet aggregation was assessed immediately after blood draw; however with increasing sample storage, even direct thrombin inhibitors and heparin affected aggregation [39].

Observation of thrombus formation ex vivo in perfusion chambers showed that compared to native blood, citrate significantly inhibited platelet attachment kinetics, reduced adhesion, nearly abolished platelet aggregation and reduced thrombus stability [40]. Citrated blood did not reliably reflect fresh whole blood coagulability measured using thromboelastography [41–43]. Because of variations in calcium concentration, recalcification of citrate-anticoagulated blood does *not* yield a reliable depiction of natural blood coagulation dynamics [34, 44].

Perhaps the most important problem with use of citrate anticoagulation is that at the very low resultant plasma iCa concentration, activated platelets do not generate thrombin. Thrombin generation by activated platelets is one of the most important drivers of thrombus growth and stabilization. Since thrombin is the most potent platelet agonist, locally-generated thrombin accelerates platelet aggregation and through the formation of a stabilizing fibrin network, renders the thrombus resistant to dislodgement by arterial pressure. Plasma iCa concentration in citrated blood is ~ 0.1 mM while the threshold concentration of free calcium required for thrombin generation is 0.25 ± 0.05 mM [45], such that in citrate-anticoagulated blood, activated platelets do not generate thrombin. Variation in iCa concentration of citrate-anticoagulated plasma may be responsible for the high variation, low reproducibility and low sensitivity to coagulation deficiencies observed with thrombin generation tests [34, 44].

Slightly elevated or high-normal iCa concentrations (but still within the normal range) were independently associated with increased levels of fibrinogen and a higher risk of cardiovascular disease [46]. Furthermore, chelation of calcium leads to conformational changes in coagulation factors V and VIII, resulting in loss of procoagulant activity. Calcium is also required at many points within the coagulation cascade, the tenase and prothrombinase complexes to function, and also mediates the binding of FXa and FIXa to the phospholipid surface of platelets and may all be impacted by fluctuations caused by citrate and recalcification.

#### Alternative anticoagulants

The aforementioned adverse effects of citrate highlight the need to consider alternative anticoagulants in POC PFTs. However, heparin enhances primary aggregation and induces aggregation by potentiating platelet release reaction. Akin to that observed in citrated blood, platelets tend to clump in samples collected into unfractionated or low molecular weight heparin, precluding the use of these as anticoagulants in PFTs [47–49]. Because of the pivotal role of thrombin in thrombosis, the use of thrombin antagonists (hirudin, PPACK, direct thrombin inhibitors) would make any PFT highly nonphysiological and use of these anticoagulants is limited to research. Native, non-anticoagulated blood should therefore be used.

### Static versus pathological flow conditions

Arterial flow and haemodynamic forces have dramatic impact on thrombus formation, coagulation and lysis of an arterial thrombus [21, 50]. In a severely stenotic artery, thrombus formation mainly results from shear-dependent platelet aggregation [23–25], with soluble agonists released from platelets playing a secondary role in stabilizing formed aggregates [51, 52]. By extending the range of shear at which thrombi are formed, shear gradients (stenoses) dramatically change platelet thrombi formation compared to constant base shear alone. Furthermore, individual healthy donors displayed quantifiable differences in extent/formation of thrombi within varying shear gradients [53].

The effect of high shear forces on platelets in initiating, propagating and stabilizing arterial thrombi is two-fold. At pathological shear rates (threshold > 10,000 s^−1^), conformational extension of vWF and its interaction with platelets initiates thrombus formation by causing an explosion of thrombus growth with rapid platelet aggregation, independent of activation [54, 55].The importance of high shear in initiating a thrombotic reaction in a severely stenotic artery is exemplified in a recent study which showed that amongst patients with > 70% luminal stenosis of the internal carotid artery, those with a shear rate in excess of 8000 s^−1^ were 12 times more likely to experience cerebrovascular ischemic events than those with lower shear rates [56].

In vivo or in extracorporeal flow chambers with circulating flow loops, the repeated exposure of platelets to lower shear rates (< 10,000 s^−1^) during recirculation, so called “platelet hammering”, can trigger persistent platelet aggregation. However, in ex vivo devices where platelets are exposed very briefly to high shear while passing through the stenotic region only once, stable aggregate formation requires exposure to much higher shear (> 20,000 s^−1^) [52].

Blood flow and haemodynamic forces also affect localized thrombin generation, fibrin formation and structure. Upon activation by collagen, thrombin or high shear, a subpopulation of platelets externalize the procoagulant phospholipid phosphatidylserine (PS) and release PS-exposed microvesicles, resulting in localized thrombin generation and fibrin formation [57, 58]. In contrast, platelet activation by ADP, vWF, or thromboxane A_2_ does not generate procoagulant platelets. Some studies report that platelet pretreatment with aspirin or a P2Y_12_ inhibitor has minimal effect on the generation of procoagulant platelets and their thrombin-generating potential [59]. In the absence of plaque rupture and abundance of TF, most thrombin generated in and around the growing thrombus is formed in situ by this mechanism. Localized thrombin-generation rate within the growing thrombus and on thrombogenic surfaces has been described as a function of shear stress/rate [59–61]. High thrombin concentrations produce dense networks of fibrin fibers, rendering the thrombus relatively resistant to fibrinolysis. In contrast, low thrombin concentrations produce coarse networks of fibrin fibers, and such thrombi are relatively susceptible to fibrinolysis [52].

The importance of testing blood under pathologically-relevant flow conditions is further reflected by the lack of effect of aspirin at high shear rates. Although the antiplatelet effects of aspirin are well recognized, at shear rates corresponding to an 80% arterial luminal stenosis, aspirin did not affect either the growth rate of thrombus or the thrombotic occlusion time [62, 63]. This is further exemplified by the observation that antiplatelet agents in routine clinical use have very limited efficacy in modulating the *hypershear*-mediated platelet activation that is associated with mechanical circulatory support [64]. Individuals with diabetes have heightened platelet reactivity, resulting in a pro-thrombotic state [65]. This is partly attributable to endothelial dysfunction, resulting in reduction in nitric oxide and prostacyclin synthesis and increased release of vWF. Hyperglycemia has been shown to enhance shear stress-induced platelet activation, which may explain why individuals with diabetes respond less well to standard antiplatelet therapy. It has been suggested that optimal monitoring of antiplatelet therapy using vWF/high shear PFT may provide a better guide to adjustment of antiplatelet treatment in diabetic individuals than other PFTs [66].

Most POC PFTs do not employ flowing blood under high shear or shear gradient, making these not only nonphysiological but notably, since platelet deposition onto reactive surfaces induced by shear gradients is not prevented by aspirin, clopidogrel or thrombin inhibitors so the effect of these drugs may be overestimated by low-shear tests [23]. Whilst some employ static conditions (VerifyNow, Multiplate, ROTEM/TEG), others employ slightly higher shear (such as the PFA), whilst some employ pathological high shear conditions (Global Thrombosis Test).

### Assessment of endogenous fibrinolysis

The clinical outcome following an ACS is determined by the interplay between pro-thrombotic and endogenous thrombolytic activities in blood [19]. When the balance is altered in favour of platelet activation and/or coagulation, or if endogenous fibrinolysis becomes less efficient, thrombosis can occur, resulting in vessel occlusion [19, 52]. Assessment of the true global thrombotic status therefore requires measurement of not only platelet reactivity and coagulation, but also of the endogenous fibrinolytic activity. Recent evidence shows that endogenous fibrinolysis is significantly impaired in a number of patients with ACS and is a strong and independent predictor of recurrent adverse cardiovascular events [7, 67, 68].

The intrinsic clot lysis theory was put forward 60 years ago by Alkjaersig, Fletcher, and Sherry in 1959. As such, endogenous thrombolysis is regarded as an intrinsic process, where activators, inhibitors and modulators of fibrinolysis are already localized within the developing thrombus. Major factors distinguishing lysis of an arterial thrombus from plasma or whole blood clot lysis are arterial flow, the presence of platelets, locally-generated thrombin and adherent leukocytes. While it is fibrinolysis which plays the major role in endogenous thrombolysis, the stability of the thrombus in withstanding dislodgement by arterial flow [52] and clot retraction [52, 69] are also important determinants of lasting vessel occlusion.

Blood flow is necessary to accurately assess endogenous thrombolysis. Flow localizes plasminogen during thrombus formation [70] and promotes thrombolysis by enhancing the transport, binding and penetration of plasminogen activators into the thrombus core [71, 72]. Flow promotes accumulation and adherence of leucocytes in the thrombus, where they are key modulators of fibrin structure and lysis. Both monocytes and neutrophils can modulate the activity of the fibrinolytic pathway and impact the susceptibility of formed fibrin to fibrinolysis [73]. Neutrophils can cause hyperfibrinolysis by releasing the proteolytic enzyme elastase, but under different conditions (“fibrinolysis shutdown”) inhibit fibrinolysis by degrading plasminogen [74]. Platelets are the main modulators of thrombolysis [75, 76] by accumulating plasminogen from the circulation in the core of thrombus and supplying the major inhibitors PAI-1, alpha 2-antiplasmin, protease nexin-1 and thrombin activatable fibrinolysis inhibitor (TAFI). Thrombin generated locally by activated platelets plays the major role in the resistance of thrombus to lysis. In addition to determining the size of fibrin fibres with promotion of thin, less porous and more compact fibrin fibre network, locally-generated thrombin has a bidirectional function in endogenous fibrinolysis by facilitating fibrinolysis via inactivation of PAI-1 and through inhibition of fibrinolysis by inactivating pro-urokinase or activation of TAFI.

As the above factors are not present in the static systems commonly used to assess plasma or whole blood fibrinolysis, such tests are not relevant to in vivo endogenous thrombolysis and not suitable to assess endogenous thrombolytic potential in individual patients. Only a test which can form autologous, platelet-rich thrombi from native blood under ex vivo pathological flow conditions and detect the restoration of flow after occlusive thrombus formation can claim to have pathological relevance. Such animal models of thrombolysis have been used for some time in research [77] but the principle of these techniques did not manifest in POC test, although early data are promising with the Global Thrombosis Test [19, 52]. It is proposed that high shear/vWF-based tests may provide more useful information on adjusting antiplatelet therapy in individual patients with atherosclerosis.

### The ideal point-of-care thrombosis test

Based on the importance of the need to reproduce a physiological milieu and the need to avoid the complications of anticoagulation, the ideal POC PFT should employ non-anticoagulated whole blood, which necessitates immediate assessment and avoidance of storage. The test should not only assess platelet activation and aggregation but reflect whole blood thrombus formation. To replicate arterial thrombosis, the test should employ conditions of high shear gradient, during which thrombin is the driving force for thrombus formation and also the main determinant of thrombus stability. Whilst the contribution of the endothelium is difficult to replicate, contact activation due to the artificial surfaces of PFTs contributes toward simulation of coagulation activation by endothelial disruption.

Whilst complex ex vivo laboratory models in use and in development, use of animal models of atherosclerosis, flow circuits, platelet-like particles or nanovesicles may better reflect arterial thrombosis than POC tests [78, 79], such techniques do not replace the need for a POC, clinically-friendly test of thrombosis.

The ideal test should employ blood that is flowing under constant arterial pressure, which is subjected to a site of pathological high shear gradient, that leads to occlusive thrombus formation. Such a test should not only assess thrombus growth, but also the susceptibility of the thrombus to endogenous thrombolysis, as well as stability and propensity to embolization.

## Conclusion

Recognition of the importance of altered flow conditions, hemodynamic forces, procoagulant activity of platelets and endogenous thrombolytic activity is not reflected in most POC PFTs currently in mainstream use. Most tests still rely on specific agonist-induced platelet aggregation using citrate-anticoagulated blood under static conditions, while the significance of high flow, shear and locally-generated thrombin is ignored. To reflect physiological processes, POC tests should assess non-anticoagulated blood at high shear, to reflect and assess the dynamics of thrombus formation, platelet reactivity and thrombus stability, as well as the rate of endogenous thrombolysis of the occlusive thrombus. Future tests should incorporate all these features and physicians employing POC PFTs should chose the most physiological tests to best reflect the thrombotic and thrombolytic status of patients, and to better assess response to pharmacotherapy.

## Abbreviations

ACS: Acute coronary syndrome
ADP: Adenosine diphosphate
iCa: Ionized calcium concentration
HTPR: High on-treatment platelet reactivity
NETs: Neutrophil extracellular trapsV
PAI-1: Plasminogen activator inhibitor
PFT: Platelet function test
POC: Point-of-care
PS: Phospholipid phosphatidylserine
TAFI: Thrombin-activatable fibrinolysis inhibitor
TF: Tissue factor
TFPI: Tissue factor pathway inhibitor
vWF: Von Willebrand factor
